# Anaplasmosis in the Amazon: diagnostic challenges, persistence, and control of *Anaplasma marginale* and *Anaplasma phagocytophilum*

**DOI:** 10.3389/fvets.2025.1571694

**Published:** 2025-05-14

**Authors:** Jhorsan David Mauri Pablo, Jakson Jacob Chuquimia Del Solar, Elthon Thomas Hinojosa Enciso, Richard Costa Polveiro, Dielson da Silva Vieira, Eduardo Milton Ramos Sanchez, William Bardales Escalante, Jorge Luis Maicelo Quintana, Rainer Marco Lopez Lapa

**Affiliations:** ^1^Instituto de Investigación en Ganadería y Biotecnología, Facultad de Ingeniería Zootecnista, Agronegocios y Biotecnología, Universidad Nacional Toribio Rodríguez de Mendoza de Amazonas, Chachapoyas, Peru; ^2^Laboratorio de Fisiología Molecular, Instituto de Investigación en Ganadería y Biotecnología, Facultad de Ingeniería Zootecnista, Agronegocios y Biotecnología, Universidad Nacional Toribio Rodríguez de Mendoza de Amazonas, Chachapoyas, Peru; ^3^Faculdade de Medicina Veterinária e Zootecnia (FMVZ), Universidade Federal de Uberlândia (UFU), Uberlândia, MG, Brazil; ^4^Department of Basic Medical Sciences, College of Veterinary Medicine, Purdue University, West Lafayette, IN, United States; ^5^Facultad de Medicina, Universidad Nacional Toribio Rodríguez de Mendoza de Amazonas, Chachapoyas, Peru; ^6^Facultad de Ingeniería Zootecnista, Agronegocios y Biotecnología, Instituto de Investigación en Ganadería y Biotecnología, Universidad Nacional Toribio Rodríguez de Mendoza de Amazonas, Chachapoyas, Peru; ^7^Facultad de Ingeniería Zootecnista, Agronegocios y Biotecnología, Universidad Nacional Toribio Rodríguez de Mendoza de Amazonas, Chachapoyas, Peru

**Keywords:** anaplasmosis, *Anaplasma marginale*, *Anaplasma phagocytophilum*, Amazon basin, tick-borne pathogens

## Abstract

Anaplasmosis remains a significant threat to livestock production in tropical regions, particularly in the Amazon basin, where ecological complexity and limited veterinary infrastructure challenge effective disease management. This review focuses on *Anaplasma marginale* and *Anaplasma phagocytophilum*, the primary species associated with bovine and granulocytic anaplasmosis, respectively. We examine the current state of diagnostic tools, highlighting the limited accessibility of molecular techniques in rural settings and the emerging but underutilized potential of technologies. Persistent infection and antigenic variation are explored as major obstacles for disease eradication and vaccine development. Although live attenuated and inactivated vaccines are in use for *A. marginale*, none provide sterilizing immunity, and no commercial vaccines exist for *A. phagocytophilum*. The review evaluates recent advances in recombinant antigens, chimeric constructs, and genetically attenuated strains, as well as future directions involving multiepitope design, novel adjuvants, and next-generation vaccine platforms. Additionally, we assess the role of tick control in disease prevention and emphasize the importance of integrated strategies in regions like the Amazon. Together, these findings underscore the need for context-specific solutions that address the ecological and epidemiological complexity of anaplasmosis in the Amazon basin.

## 1 Introduction

Livestock diseases pose a significant challenge to animal health and threaten the economic stability of agricultural sectors worldwide. Effective management of these diseases is crucial for ensuring the well-being of livestock and maintaining the productivity and profitability of the agricultural industry.

The largest cattle population in South America is found in Brazil (238,626,442 head), followed by Argentina (54,460,799 head), Colombia (27,239,767 head), Paraguay (13,801,993 head), Uruguay (11,400,963 head), and Peru (5,160,000 head). Cattle rearing is a significant economic activity in these countries, which are major players in the agribusiness sector (1). The total bovine population in South America is approximately 384 million, accounting for 25% of the world’s cattle inventory. This makes the region a major contributor to the global beef market, playing a vital role in beef exports (2).

Among the diseases that significantly affect livestock production is anaplasmosis, which disrupts production and reduces yields in milk and meat. Additionally, this disease incurs high costs associated with veterinary care and treatment (3). Anaplasmosis is a vector-borne disease caused by bacteria of the genus *Anaplasma*, obligate intracellular pathogens belonging to the family Anaplasmataceae. These bacteria infect a wide range of vertebrate hosts, including both wild and domesticated mammals, as well as some birds. Their transmission typically occurs through tick vectors, contributing to the spread and persistence of the disease in diverse ecological settings. Anaplasmosis has been reported in cattle, buffalo, sheep, goats, and some non-domestic ruminants (4–6). It is primarily transmitted by ticks of the genera *Ixodes*, *Rhipicephalus*, and *Dermacentor* (7). Key species include *Anaplasma marginale* (bovine anaplasmosis), *Anaplasma phagocytophilum* (granulocytic anaplasmosis in humans and animals), *Anaplasma platys* (infects platelets in dogs), *Anaplasma ovis* and *Anaplasma capra* (affecting small ruminants) (5, 7, 8).

In the Amazonian region, anaplasmosis represents a complex veterinary and zoonotic concern (9), where not only biodiversity and climate play a role, but also cultural practices such as animal trade, uncontrolled livestock movement between regions, and limited implementation of anti-tick control programs contribute to the maintenance of these pathogens in local herds (10).

**Figure 1:**
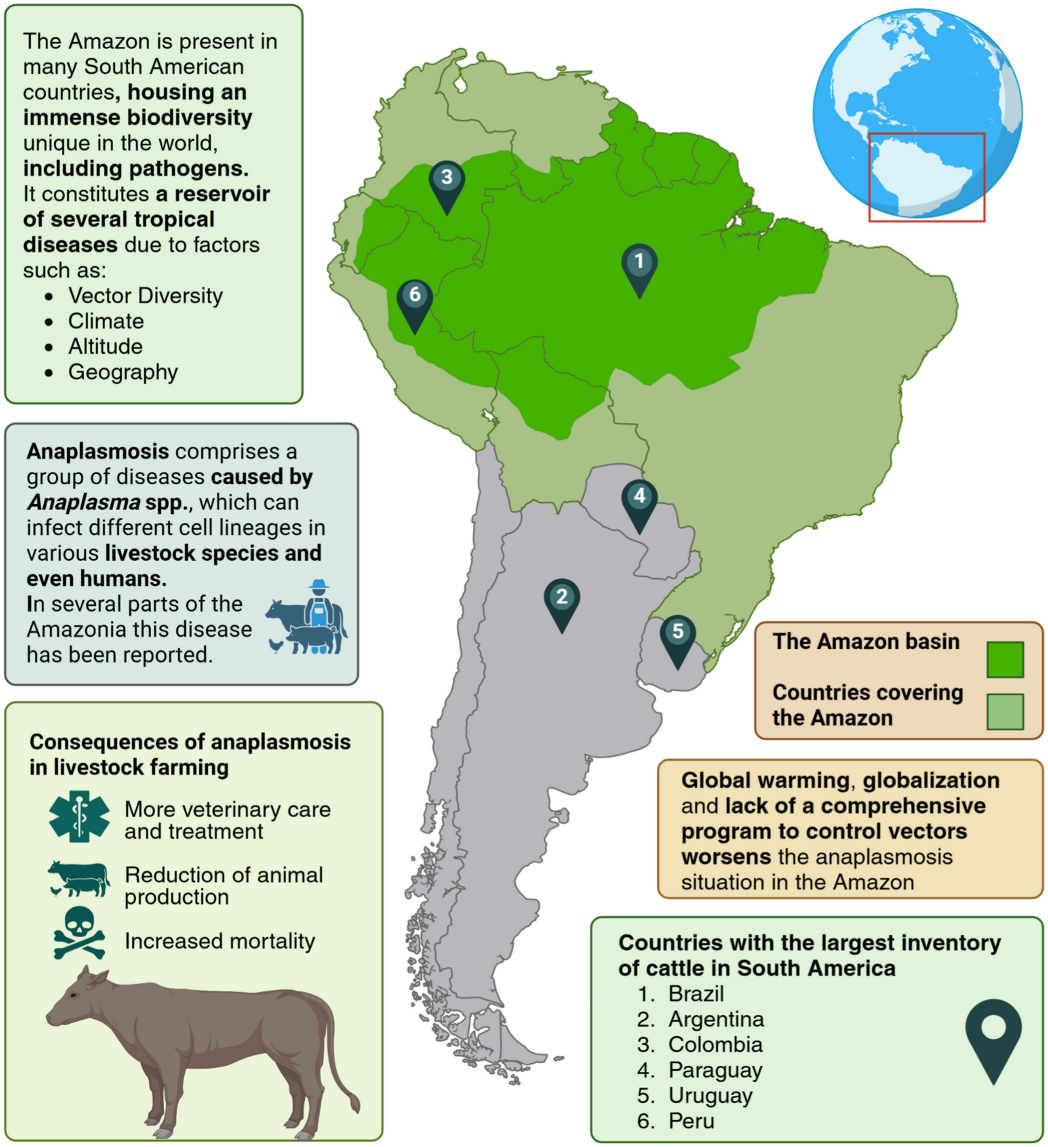
Epidemiology of anaplasmosis in the Amazon: a vector-borne disease affecting livestock. Created in https://BioRender.com.

The Amazon basin covers an area of about 7 million km^2^, with Amazon forests comprising approximately 5.3 million km^2^–40% of the world’s tropical forest area (11) (Figure 1). Recent studies suggest unique transmission cycles in wild animals, as evidenced by novel genetic variants of *Anaplasma* in the Amazon basin. For instance, *Candidatus Anaplasma sparouinense*, identified in French Guiana, is an emerging species associated with human infections and sloths (12).

In the Brazilian Amazon, the presence of *A. marginale* and *A. phagocytophilum* has been confirmed in both domestic and wild animals, highlighting the potential risk of zoonotic transmission (12, 13). The region’s high biodiversity of vectors, particularly ticks and tropical climate, facilitates the propagation of these pathogens, making anaplasmosis a disease of significant concern (12). Additionally, other types of transmission have been reported, apart from vectors, such as transplacental in cattle (14) and possibly nosocomial (15, 16).

In Peru, anaplasmosis causes substantial economic losses, particularly in the Peruvian Amazon. A study in the districts of Omia and Molinopampa in Amazonas reported a 67% prevalence of *A. marginale* in Simmental cattle, emphasizing its negative impact on milk and meat production (17). Small and medium-scale producers are especially vulnerable, as the associated veterinary and treatment costs further strain their resources. These challenges are exacerbated by geographic factors, such as an altitude of 1701 to 2000 meters above sea level, which influences the disease’s prevalence (17).

In South America, the combined economic losses from anaplasmosis and babesiosis are estimated at $875 million annually, with Brazil alone reporting losses of $3.24 billion due to *Rhipicephalus microplus* and *A. marginale*. In Mexico, the economic toll is approximately $942.23 million, excluding associated costs such as antibiotics and treatments (18).

## 2 Anaplasmosis in livestock: clinical symptoms and impacts

**Figure 2:**
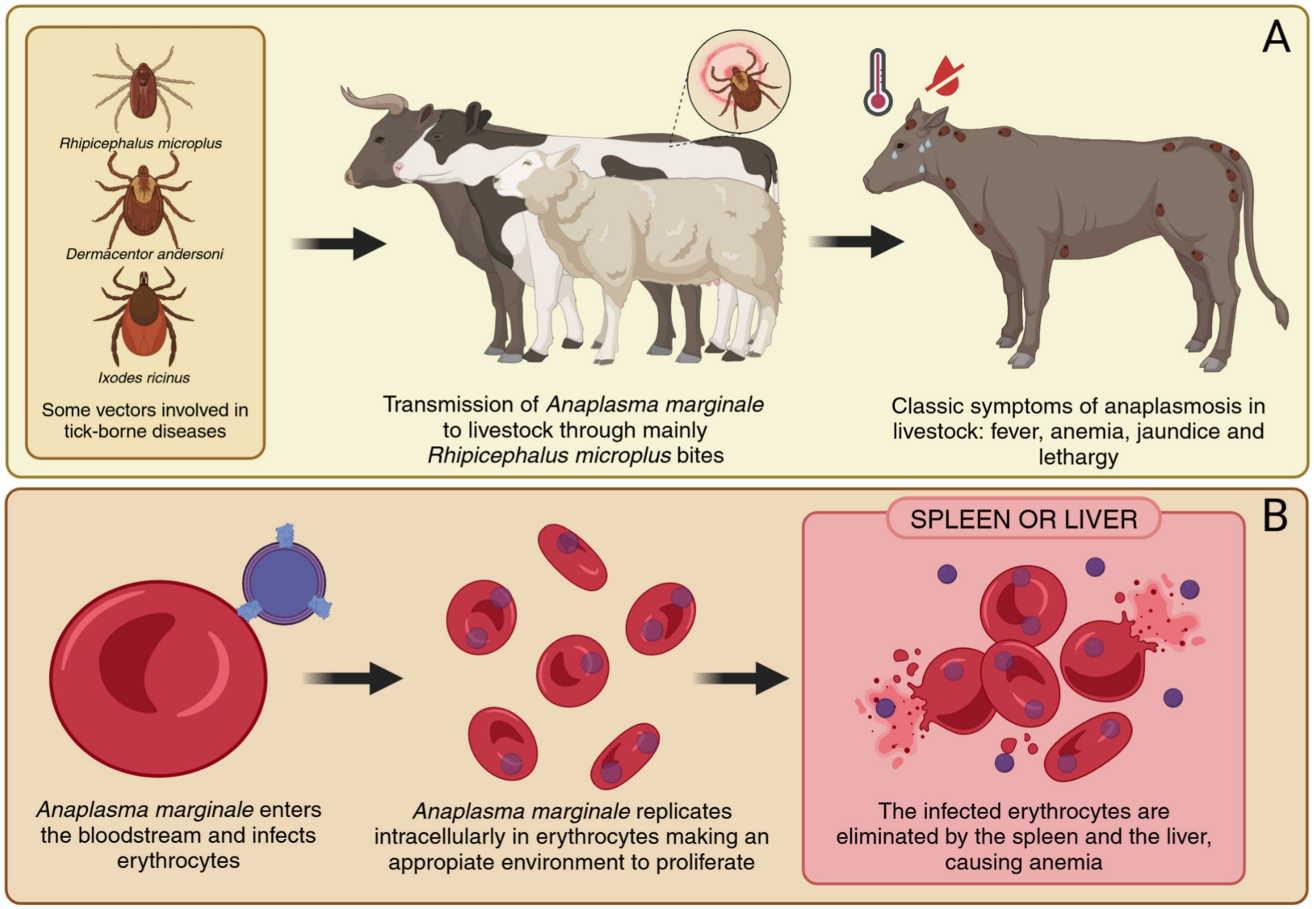
Transmission and pathogenesis of anaplasmosis in livestock caused by *A. marginale*. **(A)** Transmission of *A. marginale* through tick bites, mainly by *Rhipicephalus microplus*, and onset of clinical symptoms in livestock. **(B)** Progression of *A. marginale* infection, including immune evasion, replication in erythrocytes, and destruction of infected erythrocytes. Created in https://BioRender.com.

*A. marginale* is the most common pathogen in bovines, primarily infecting erythrocytes and causing severe anemia, fever, jaundice, lethargy, and weight loss (19). These clinical symptoms lead to significant economic losses due to decreased productivity and increased mortality, particularly in regions like the Amazon basin, where small-scale livestock producers face limited access to veterinary care. The transmission of *Anaplasma* spp. occurs mainly through tick bites, with vectors such as *Rhipicephalus microplus*, *Dermacentor andersoni, Rhipicephalus sanguineus*, and *Ixodes ricinus.* In the Amazon, the high biodiversity of wildlife also serves as reservoirs, complicating disease management in livestock populations (20, 21) (Figure 2A).

The pathogenesis of anaplasmosis involves sophisticated interactions between Anaplasma pathogens and the host immune system. Several species of the genus Anaplasma are known to infect different hosts and exhibit distinct cellular tropisms. *A. marginale* primarily affects cattle and invades erythrocytes, while *A. phagocytophilum* has a broad host range. Including humans, dogs, horses, cattle, sheep, goats, deer, and small mammals, and specifically targets neutrophils and other granulocytes. *A. platys* infects dogs and parasitizes platelets, whereas *A. ovis* is found in sheep and goats, also targeting erythrocytes. *A. centrale*, a naturally less virulent species used as a live vaccine, infects bovine erythrocytes. In contrast, *A. bovis* has been detected in cattle, buffalo, goats, deer, and dogs, and exhibits a tropism for monocytes (22).

In *A. marginale*, major surface proteins (MSPs) are located in the outer membrane of the bacterium, allowing direct interaction with host cells. Specifically, MSP1a and MSP1b mediate adhesion to bovine erythrocytes and to the gut epithelial cells of tick vectors, facilitating both infection and transmission (23). Additionally, MSP1a plays a key role in strain characterization and epidemiological studies, as its tandem repeat sequences vary among isolates and reflect geographic and biological diversity (23).

*A. marginale* evades immune detection through antigenic variation, particularly in MSP2 and MSP3, which are encoded by multigene families subject to recombination and sequential expression. This mechanism enables the pathogen to persist within the host by continuously altering its surface antigens, escaping recognition by the immune system (24). In contrast, MSP4 is a conserved protein encoded by a single gene, serving as a stable molecular marker for species identification but lacking evidence of antigenic variability (25). This intracellular survival, coupled with the ability to downregulate pro-inflammatory cytokines and manipulate immune cell functions, creates a favorable environment for the pathogen’s replication and survival (26, 27). These mechanisms not only facilitate chronic infections but also pose significant challenges for vaccine development and therapeutic strategies (Figure 2B).

In the Amazon region, the warm climate and high biodiversity create ideal conditions for the proliferation of vectors, including ticks of the genus *R. microplus* (formerly *Boophilus microplus*) and *Dermacentor*, which are key vectors for *A. marginale*. These ticks are also vectors for other bacteria, adding complexity to the control of tick-borne diseases (28).

In the Brazilian Pantanal, part of the Amazon biome, *A. marginale* is highly prevalent among beef cattle, with studies indicating 72.2% seropositivity using iELISA and 56.7% positivity via qPCR targeting the msp1β gene (29). This prevalence highlights the role of the tropical climate, which favors the proliferation of tick vectors such as *R. microplus* and *Amblyomma sculptum*. Cows acted as chronic carriers, perpetuating the pathogen, while male calves sold to other regions posed a risk of disseminating diverse strains of *A. marginale*. These findings underline the disease’s persistence in tropical regions and its economic implications.

In northeast Brazil, specifically in the region of Médio Mearim, Maranhão state, *A. marginale* was detected in 95.32% of the dairy cattle using qPCR targeting the gene msp1β, while 81.34% of the samples were seropositive in iELISA assays using the recombinant protein MSP5. The high seropositivity in cows compared to calves suggest an cumulative exposure to the pathogen through time. These findings reflect the high prevalence of *A. marginale* in Amazon-Cerrado transition zones, where the environmental conditions enhance the proliferation of vectores like *R. microplus* (30).

## 3 The role of molecular techniques in advancing anaplasmosis detection and control

Molecular techniques have significantly advanced the detection and control of anaplasmosis by enabling precise identification of Anaplasma species, even in subclinical cases (31, 32). Tools like PCR, qPCR, and LAMP enhance diagnostic accuracy and support surveillance in endemic areas. Their strategic application improves early intervention and disease management. Despite challenges, they bridge research and practical control efforts (33).

The range of diagnostic techniques for *Anaplasma* detection spans from conventional PCR methods to more advanced genomic tools. While PCR-based assays are commonly used in research and reference laboratories due to their sensitivity and specificity, their routine application in bovine anaplasmosis diagnosis remains limited in many endemic regions because of cost and infrastructure constraints. In field settings, diagnosis still often relies on clinical signs and microscopy. On the other hand, whole-genome sequencing and metagenomic approaches are primarily restricted to experimental or epidemiological studies, where they offer valuable insights into pathogen diversity and host–vector dynamics (34). Nevertheless, the adoption of these molecular tools in routine veterinary practice remains challenging, and their implementation is often restricted to government programs or academic research.

In contrast, serological tests such as ELISA, complement fixation, and card agglutination are currently the most accessible and widely used diagnostic methods for bovine anaplasmosis. These tests provide cost-effective and practical alternatives for detecting exposure to *Anaplasma*, especially in rural or resource-limited settings, and continue to be essential components of surveillance and herd-level screening.

Moreover, the integration of molecular diagnostics into epidemiological frameworks has enhanced the ability to detect subclinical infections and understand pathogen dynamics within ecosystems (35). These advancements enable early intervention strategies and contribute to the formulation of targeted control measures, ultimately reducing the burden of anaplasmosis on animal health, productivity, and public health (36). The versatility and adaptability of molecular techniques underscore their critical role in bridging scientific research and practical solutions for disease management (Table 1).

**Table 1:** Comparative overview of diagnostic techniques for anaplasmosis: global perspectives on principles, applications, and advances.

Technique	Principle	Advantages	Disadvantages	Sensitivity	Application	References
PCR	Amplification of specific DNA sequences via thermal cycling	Accurate amplification and detection of chronic or subclinical infections	Requires specialized equipment and thermal cycling that is difficult to obtain in rural areas of the Amazon	Sensitivity of 73.2% and specificity of 89% in caprines and ovines (*Anaplasma* spp.)	Initial diagnosis of *A. marginale* infections in cattle, caprines and ovines	(35, 37, 40, 44–46)
qPCR	Quantification of DNA in real-time using fluorescent probes	Allows precise quantification of bacterial load in real-time. Is a highly sensitive method for monitoring infection.	More expensive than standard PCR, requires specific probes. If standards are not well-defined, the pathogenic load may be overestimated	Detection of 100% (in 10 copies) and 70% (5 copies) (*A. phagocytophilum*). 10 times superior to LAMP (*A. marginale*)	Epidemiological studies and real-time monitoring of infection and treatment progress by *Anaplasma*	(29, 30, 42, 43)
Nested PCR	Two rounds of amplification using external and internal primers for higher specificity	Higher sensitivity and specificity compared to standard PCR	More complex and time-consuming than standard PCR	Detection from 18 to 100% at 28–38 days post-inoculation (*A. centrale*)	Detection of infections in samples with low DNA content	(37, 40, 42, 47)
LAMP	Isothermal amplification of DNA at a constant temperature	Inexpensive, easy to use, with fast and visible results that do not require complex equipment or thermal processes	Lower sensitivity compared to qPCR. It requires a more complicated primer validation and design process than PCR-based methods	Detection 100 times greater than standard PCR tests (*A. marginale*)	Quick, affordable, and easy-to-do diagnosis in low-resource and rural areas	(44, 45)
ELISA	Detection of antibodies in serum using specific antigens	Highly specific and sensitive, detects early infections. Simple and accessible without complex equipment	Only measures exposure to the pathogen, not active infection. Requires good characterization and availability of antigens	Sensitivity of 77.7–98.2% (*A. marginale*) and 86.3–96.6% (*A. centrale*). Specificity of 97.4–98.7%. In caprines and ovines, sensitivity of 91.9% and specificity of 86.9% (*Anaplasma* spp.)	Epidemiological surveillance and serological diagnosis	(30, 46, 47)
HTS	Massive parallel sequencing of DNA fragments	Detects known and emerging pathogens, allows metagenomic analysis	Expensive, requires advanced equipment and data analysis	High sensitivity, capable of detecting multiple pathogens simultaneously	Genomic studies, identification of variants and resistance genes	(48–51)
Microarrays	Simultaneous detection of multiple pathogens in a single test	Allows epidemiological surveillance, adaptable to new pathogens	Initially expensive, requires specialized equipment and personnel for analysis and detection	Sensitivity and specificity of 100% (*A. marginale*). Detection in samples 10–1,000 times less concentrated than PCR (*Anaplasma* spp.)	Diagnosis of multiple infections, especially in endemic regions	(52–56)
HRM	Melt curve analysis post-PCR to detect genetic variants without sequencing	High resolution, useful for epidemiological studies	Requires specific DNA preparation and equipment, and may not have high enough resolution	High sensitivity for genetic variation detection	Rapid diagnosis and detection of genetic variants in infections	(57)
GWAS	Identification of genetic variants associated with traits through the analysis of the entire genome of a population	Identification of specific *loci* associated with important phenotypic traits	Requires very large sample sizes to detect associations with statistical power	High sensitive to common genetic variants but has limitations in detecting rare variants	Identifies genetic variants associated with the immune response of cattle to anaplasmosis infection	(58–60)
Western blot (WB)	Detection of specific proteins via antibodies	Enables detailed evaluation of host immune response	Requires specialized equipment and technical expertise	High specificity for major surface proteins	Diagnosis of *Anaplasma* spp. infections	(57, 61)
MALDI-TOF MS	Identification of microorganisms via protein pattern analysis	Rapid, accurate pathogen identification, including vectors	Requires costly equipment and sample preparation	High specificity	Identification of pathogens and vectors in epidemiological studies	(62, 63)
RPA-CRISPR/Cas12a	Isothermal DNA amplification via RPA combined with CRISPR-based detection	Highly sensitive, visual results possible, suitable for low-resource areas	Still under development for certain applications	Detects from 4 copies/μL of DNA	Rapid, accessible diagnosis in rural areas with visual result	(25)
IFA	Antibody marked with fluorescence is then added to reveal the presence of antibodies under a fluorescence microscope	High sensitivity and specificity	Costly, time-consuming, and subject to observer bias due to its multi-step process and subjective interpretation	Sensitivity and specificity of 100% in caprines and ovines (*Anaplasma* spp.)	Detects *A. phagocytophilum* in ovine samples	(64)
Agglutination Test	Detects antibodies through the formation of visible agglutinates	Simpler and more cost-effective, and it is effective as a quick screening method	Lower sensitivity and specificity can result in cross-reactions, leading to false positives	Sensitivity of 95.21% and specificity of 91.68% (*A. marginale*)	Detects antibodies against *A. marginale*	(65)

The references cited in this table are derived from studies conducted worldwide to provide a comprehensive overview of diagnostic techniques for anaplasmosis.

### 3.1 PCR, qPCR, and nested PCR

#### 3.1.1 PCR

Polymerase Chain Reaction (PCR) is an essential technique for the molecular identification of *A. marginale*, because it facilitates the amplification of specific sequences in the DNA which allows the detection of this pathogen in blood samples. This technique is especially useful in chronic or subclinical infections, in which the bacterial load may be low, which makes its detection harder by traditional methods (37). In the case of *A. marginale*, genes such as msp4 are commonly used as targets in PCR due to their high conservation and usefulness in molecular detection and phylogenetic analysis, rather than a direct role in pathogenesis (37). Other genes like 16S rRNA, msp1α, msp1β, groEL, and gltA have also been employed in PCR and qPCR assays for the detection of anaplasmosis (38–41). In Brazil, combinations of msp1α, msp1β, 16S rRNA, and msp4 have proven effective in characterizing the genetic diversity and phylogeny of *A. marginale* strains (41).

#### 3.1.2 Quantitative PCR—qPCR

qPCR is an evolution of the conventional PCR because it does not only detect but also quantifies the DNA of *Anaplasma* in real time, using fluorescent probes which allow it to observe the process in real time. An example of its application is the use of a TaqMan probe in the qPCR to detect the gen msp1b of *A. marginale* in livestock’s blood sample (30, 42). The qPCR is especially useful in epidemiologic studies and the evaluation of the efficacy of treatments because it allows the detection of sub clinic infections and quantification of the bacterial load, which is essential for the control of anaplasmosis in endemic areas (42).

To identify *A. marginale*, de Souza Ramos et al. (29) employed a multi-faceted molecular approach. qPCR targeting the msp1β gene achieved high sensitivity, while semi-nested PCR for the msp1α gene revealed 14 distinct strains, including eight novel ones. The use of tools like the RepeatAnalyzer software (43) for analyzing tandem repeats in the msp1α gene further enhanced genetic diversity assessment. Such methodologies are pivotal for surveillance and control, especially in regions like the Amazon, where environmental factors and genetic variability challenge disease management.

#### 3.1.3 Nested PCR

Nested PCR is a variant of the PCR which increases the sensibility and specificity of the conventional technique by two rounds of amplification. In the first round, external primers are used to amplify a specific region of the DNA, and in the second round, internal primers are used to amplify a fragment within the product of the first PCR. This method is particularly useful to detect *A. marginale* in samples with low load of pathogens or when the DNA quantity is limited (37). Nested PCR has proven to be effective in the identification of infections in animals and ticks, identifying species like *A. ovis* and *A. marginale* which is important in epidemiologic and disease control studies in endemic regions (37).

Standard PCR, qPCR and Nested PCR are complementary techniques in the detection of *Anaplasma* sp. Standard PCR is useful for initial detection and confirmation of infections while qPCR offers advantages in the quantification and detection of sub clinical infections, which is crucial to surveil the progression of the disease and effectiveness of the treatment (40). On the other hand, Nested PCR gives more sensibility, making it ideal for the detection of infections in samples with low load of pathogens (42).

### 3.2 LAMP (Loop-Mediated Isothermal Amplification)

Loop-Mediated Isothermal Amplification (LAMP) has demonstrated promising potential for the detection of *A. marginale*, particularly in low-resource settings. Unlike conventional PCR, which requires repetitive thermal cycling, LAMP operates at a constant temperature, reducing the need for costly equipment such as thermocyclers (44). However, its application to bovine anaplasmosis remains limited, with only a few published studies to date. Further validation and broader adoption are needed before it can be considered a routine diagnostic tool.

The principle of LAMP involves the use of 4–6 primers that recognize distinct sequences within the target DNA. This not only enhances specificity but also accelerates the amplification process, enabling DNA detection in less than an hour. For *A. marginale*, LAMP assays have been developed using genes such as msp5 and msp1b, which are highly specific to this species (45). Compared to conventional PCR, LAMP offers the additional advantage of allowing visual detection of amplified products, thereby eliminating the need for supplementary techniques like gel electrophoresis (45).

Recent studies have demonstrated that LAMP can be as or even more sensible than conventional PCR for the detection of infections by *A. marginale*. Giglioti et al. (44) discovered that LAMP can detect from 211 copies/μL of *A. marginale* DNA, while PCR can detect from 21 copies/μL, which means that although LAMP is highly specific, it has less sensibility than qPCR (44). However, LAMP is still extremely useful in low-income areas due to its simplicity and quick detection without specialized machines.

Another study by Ganguly et al. (45) compared the efficacy of LAMP and conventional PCR in infected livestock samples in Gujarat, India. This study demonstrated that LAMP detected *A. marginale* in 22.14% of the samples, in comparison with 15.36% detected by PCR, which suggests a higher sensibility in LAMP technique (45). Also, LAMP has demonstrated a high specificity, given that no cross-amplification was observed with other hemiparasites like *Babesia bigemina* or *Theileria annulate*.

### 3.3 ELISA (enzyme-linked immunosorbent assay)

ELISA is a serologic technique widely used for the detections of antibodies against *A. marginale* in livestock, and it’s one of the most common tools for the surveillance and diagnostics of anaplasmosis. This technique detects specific antibodies in the serum of animals, giving an indirect measure of the exposition to the pathogen (46).

There are different variants of ELISA, including competitive ELISA (cELISA) and indirect ELISA (iELISA). cELISA is one of the most used for antibodies detection against *A. marginale* due to its high sensibility and specificity. In this assay, monoclonal antibodies directed against Major Surface Proteins like MSP5, which is highly conserved in *A. marginale*, are used (30, 46). The antibodies that are in the serum of the animals compete with a reference antibody that is marked to bind the antigen, which allows the detection of infections even in the early stages (46).

A more recent advancement is the Double Antibody Sandwich ELISA (dasELISA), which has proven effective in differentiating between animals infected with *A. marginale* and those vaccinated with *A. centrale*, a related but less virulent species commonly used in vaccines worldwide (47). This method employs two variants of the MSP5 protein, one specific to *A. marginale* and another to *A. centrale*, to capture and detect specific antibodies in the serum dasELISA has demonstrated not only high sensitivity and specificity but also earlier detection compared to conventional ELISA methods (47).

In comparative studies, cELISA has demonstrated a sensibility of 91.9% and a specificity of 86.9% in the diagnosis of *Anaplasma* sp. in sheep and goats, which reaffirms its use in the diagnosis of this infection in different animals (46). However, the ability of dasELISA to differentiate between species of *Anaplasma* sp. and detect infections in vaccinated animals, turns this into a useful tool in specific epidemiologic contexts.

### 3.4 High-throughput sequencing

High-Throughput Sequencing (HTS) has revolutionized the identification and characterization of pathogens, including *A. marginale*. Unlike traditional techniques, HTS allows massive and parallel DNA fragments sequencing, giving an integral vision of the microbiome and allowing an easier detection of well-known and emerging pathogens in complex samples (48, 49).

High-throughput sequencing (HTS) offers significant advantages for the metagenomic detection of tick-borne pathogens, particularly in complex infections where multiple agents may be present. Unlike conventional PCR, which targets specific sequences, HTS enables the comprehensive sequencing of all genetic material in a sample, eliminating the need for prior pathogen isolation or identification. This approach is especially useful in cases of low bacterial load or when the etiological agent is unknown. For example, in equine samples with suspected tick-borne diseases, HTS successfully identified *A. phagocytophilum* in cases that were previously PCR-negative, demonstrating its superior sensitivity and diagnostic utility (48).

Another significant advantage of HTS in metagenomic shotgun analysis is its potential to identify antimicrobial resistance genes and virulence factors, offering critical insights for the clinical management of diseases. Although the study by Subbiah et al. (48) did not detect such elements in their analysis, it emphasized that metagenomic approaches could eventually facilitate the detection of resistance determinants and virulence markers in tick-borne pathogens. This capability underscores the broader role of HTS, not only in diagnosis but also in supporting evidence-based treatment and control strategies.

Also, its high sensitivity allows the identification of strains and genetic variation of *Anaplasma* sp., which is crucial for the epidemiologic surveillance and comprehension of disease evolution. For instance, the sequencing of the whole genome of *A. marginale* has allowed the identification of variations in the MSPs, which are associated with the virulence and ability of the pathogen to evade the host’s immune system (50).

However, HTS also faces challenges, such as high costs, the need for specialized equipment and software for data analysis, and the requirement for skilled personnel to handle and interpret the data. Also, the interpretation of the results can be complex due to the high amount of generated data and the presence of environmental or background DNA in the samples (51). Despite this limitation, HTS is still a powerful tool for the identification and characterization of *A. marginale* and its use in the investigation and diagnosis of anaplasmosis is developing.

### 3.5 Microarray genotyping

The technology of microarrays has proven to be a powerful tool for detecting multiple pathogens in complex samples, including *A. marginale.* This technology allows for the simultaneous detection of different species and strains of pathogens in a single test, which is particularly useful in areas where multiple infections are common, such as endemic regions for tick-borne diseases (52).

For instance, low-density microarrays have been developed to detect bacterial pathogens and piroplasmids transmitted by ticks in African livestock. Designed to identify species within the *Anaplasma* genus alongside other pathogens, these microarrays have shown greater sensitivity than conventional techniques like PCR or Sanger sequencing, successfully detecting infections that those methods failed to identify (53).

Furthermore, the use of microarrays in diagnosing anaplasmosis not only enables the identification of active infections but also facilitates epidemiological surveillance by providing data on the prevalence and distribution of various *Anaplasma* species. Additionally, microarrays can be adapted to incorporate new and emerging pathogens, making this technology a flexible and valuable tool for safeguarding animal health in endemic regions.

While HTS and digital PCR are more sensitive and comprehensive, offering greater resolution and the ability to detect low-abundance pathogens with higher precision (54), microarrays still hold significant utility in certain contexts. Specifically, microarrays remain advantageous when the simultaneous detection of multiple pathogens is required, particularly in resource-limited settings where cost efficiency is essential (55). Their ability to screen for a wide range of pathogens in a single test, combined with relatively lower operational costs (56), makes them a valuable option for diagnostic applications in endemic regions or for large-scale epidemiological surveillance.

### 3.6 HRM (high-resolution melting) in the detection of *Anaplasma* sp.

The HMR technique has turned into a valuable technique for the detection and characterization of genetic variations in pathogens like *A. phagocytophilum*. This technique is based on the amplification of specific sequences of the DNA followed by an analysis of the fusion curves of the DNA, which allows the differentiation of different genetic variations based on differences of the sequence of nucleotide bases (57).

This technique, HRM, has been used to analyze blood samples and ticks infected with *A. phagocytophilum* in Laikipia, Kenia. This study demonstrated the efficacy of HRM to detect not only *A. phagocytophilum* but also to distinguish between different haplotypes of the pathogens in mammals and associated ticks (57). The ability of HRM to identify genetic variants in the AND sequence allows a detailed analysis of the epidemiology and transmission dynamics of *Anaplasma* species in environments where multiple hosts and vectors coexist.

### 3.7 Genome-wide association studies

The use of GWAS has opened new possibilities to understand the genetic basis of the susceptibility and resistance to diseases like anaplasmosis in livestock. GWAS allow the identification of specific loci associated with important phenotypic traits, like the resistance to tick-borne pathogens, which include *Anaplasma* sp. (58).

GWAS have demonstrated to be effective in discovering genetic variants that can influence the presence or absence of pathogens like *A. marginale* in livestock. Recent studies have identified a locus in the bovine genome that is associated with the resistance to tick-borne diseases. In a meta-analysis of GWAS done in African livestock, significant peaks were found in the chromosomes 8 and 24, which are related to the resistance to *A. marginale* and other hemiparasites transmitted by ticks (58).

These discoveries are important to develop strategies for genetic Markers Assisted Selection (MAS), which could enhance the livestock’s natural resistance to anaplasmosis. More importantly, GWAS allow the researchers to identify genetic variants that noy only are responsible for the disease resistance, but also the livestock’s ability to tolerate infections without showing severe symptoms (59).

GWAS has revealed that certain genetic variants are associated with livestock’s immune response to *A. marginale* infection. These findings could be utilized to develop genetic improvement programs, selecting animals with greater resistance to anaplasmosis, which would reduce dependence on chemical treatments and enhance the sustainability of livestock production (60). Integrating GWAS into the study of anaplasmosis provides a deeper genetic understanding of disease resistance, which is essential for developing more effective and sustainable control strategies in the livestock industry.

### 3.8 Immunoblotting, also known as Western blot

WB technique has been used for the specific detection of MSP in infections caused by *Anaplasma* spp. This technique is useful for the diagnosis of infections caused by *A. platys* and *A. phagocytophilum.* Immunoblotting allows the accurate identification of antibodies in the serum of infected animals, providing a detailed evaluation of the host’s immune response.

Mosha et al. (57) used WB along with molecular techniques to confirm the presence of *A. phagocytophilum* in small mammals and ticks, pointing out the versatility of WB in the detection of infection in multiple species.

Also, Lai et al. (61) developed a specific WB test for *A. platys*, using MSP antigens. This study highlights the importance of immunoblotting in the differentiation of *Anaplasma* species, which is crucial for the accurate diagnosis and disease control in production animals.

### 3.9 Matrix-assisted laser desorption/ionization time-of-flight mass spectrometry

MALDI-TOF MS has gained popularity for its rapid and accurate pathogen identification. It quickly compares protein mass spectra to reference databases, allowing precise identification of bacteria, fungi, and other microbes. This method is highly valuable in clinical microbiology due to its speed, precision, and cost-effectiveness in diagnosing infections, including those causing anaplasmosis. MALDI-TOF MS enables the identification of microorganisms by analyzing specific protein patterns present in samples. Recent studies have demonstrated its high specificity and sensitivity, such as in the identification of *A. phagocytophilum* in blood and tissue samples (62).

Additionally, MALDI-TOF MS has been successfully used in identifying tick species that serve as vectors for *Anaplasma* spp., which is crucial for understanding the epidemiology of tick-borne diseases. Hamlili et al. (63) demonstrated its use in identifying ticks from the genus Hyalomma and detecting *A. platys* in dromedary camels in Algeria. This ability to identify both the vector and the associated pathogen highlights the method’s versatility in epidemiological studies and diagnostics.

The high-resolution and quick analysis of MALDI-TOF MS turns this technique into a promising tool not only for the identification of pathogens but also for the disease’s surveillance and the investigation of new infection biomarkers.

### 3.10 Recombinase polymerase amplification—clustered regularly interspaced short palindromic repeats associated protein 12a

Recent advances in the detection of *A. marginale* include the use of RPA-CRISPR/Cas12a, which offers a highly sensitive and specific alternative to conventional methods like PCR. This technology combines isothermal DNA amplification via Recombinase Polymerase Amplification (RPA) with CRISPR/Cas12a-based detection, targeting the msp4 gene of *A. marginale*. Recent studies have shown that this technique can detect quantities as low as 4 copies/μl of the pathogen’s DNA, demonstrating sensitivity comparable to conventional PCR (25). A significant advantage of this technique is that its lyophilized components can be stored at room temperature, making it easier to use in rural areas and offers a good cost benefit. Additionally, it can be adapted to provide visual results through fluorescence, making it a valuable tool for the quick and affordable diagnosis of bovine anaplasmosis in rural settings (25).

### 3.11 Indirect Immunofluorescence Assay

Indirect Immunofluorescence Assay (IFA) is an important method for detecting *Anaplasma* spp. in animals, with its own advantages and disadvantages. IFA uses antigens fixed on slides, and if specific antibodies are present in the patient’s serum, they bind to the antigens. A secondary antibody marked with fluorescence is then added to reveal the presence of antibodies under a fluorescence microscope. This technique offers high sensitivity and specificity, making it very effective for detecting chronic infections like anaplasmosis. However, it is an expensive technique, requiring specialized equipment and training, and it is time-consuming due to the various steps involved. A recent study highlighted the efficacy of IFA in detecting *A. phagocytophilum* in ovine samples, with a sensitivity of 85.4% and a specificity of 100%, confirming its diagnostic accuracy (64).

### 3.12 The Agglutination Test

The Agglutination Test, on the other hand, is simpler and more cost-effective, making it widely used in field settings and in laboratories with limited resources. It detects antibodies through the formation of visible agglutinates when the patient’s serum reacts with antigens fixed on particles. While it is less sensitive than IFA, it is effective as a quick screening method. A successful example of this test is the development of a latex agglutination test for detecting antibodies against *A. marginale*, which showed good results in cattle (65).

## 4 Therapeutic and preventive strategies and recent advances

### 4.1 Antibiotics

The use of tetracyclines such as oxytetracycline and chlortetracycline remains essential in the treatment of bovine anaplasmosis. These antibiotics inhibit bacterial protein synthesis by targeting the 30S ribosomal subunit and are effective in reducing clinical signs during the acute phase. However, they do not eliminate *A. marginale* completely. In a study performed by Curtis et al. (66), cattle treated for 60 days remained asymptomatic carriers, highlighting the pathogen’s ability to persist despite prolonged treatment.

This persistence is not solely due to antimicrobial resistance. *A. marginale* exhibits several mechanisms that favor chronic infection: low bacterial loads below therapeutic thresholds, antigenic variation in msp2 and msp3 genes, and intracellular localization in erythrocytes that hinders antibiotic penetration (67). Additionally, incomplete immune responses and the risk of reinfection by vectors in endemic areas make eradication even more difficult. Importantly, while tetracyclines such as oxytetracycline and chlortetracycline remain effective for treating acute episodes, they are insufficient to eliminate carrier status, allowing the pathogen to persist within herds (66, 67).

Although no conclusive phenotypic resistance to tetracyclines has been documented in *A. marginale*, a recent study by Shahbazi et al. (68) identified the resistance-associated genes *otrA* and *otrB* in *A. marginale*, *A. ovis*, and *A. centrale* isolated from cattle blood samples. These genes are linked to ribosomal protection and efflux mechanisms, respectively, and their presence suggests a potential for the development of resistance under sustained antimicrobial pressure. To date, no treatment failures attributable to these genes have been reported, but their detection highlights the importance of continuous surveillance.

Alternative treatments include imidocarb dipropionate, which has been used with variable success, particularly in cases of co-infection. However, studies show that it does not consistently eliminate *A. marginale*, and oxytetracycline remains more effective in achieving chemosterilization in some cases (69). Therefore, imidocarb can be used as an alternative, specially when there are co infections but it does not replace the use of tetracyclines as base therapy.

In turn, doxycycline is the treatment of choice for *A. phagocytophilum* in humans and companion animals, due to its superior intracellular penetration. Although its use in ruminants is off-label, it has shown efficacy comparable to oxytetracycline in *A. ovis* infections (70).

### 4.2 Vaccines and innovative approaches

#### 4.2.1 Curent commercial vaccines against *Anaplasma marginale* and *Anaplasma phagocytophilum*

In cattle, the only commercially available vaccine is heterologous live attenuated strain of *A. centrale*, included in trivalent vaccines with Babesia in endemic countries (South Africa, Argentina, Brasil) (71). *A. centrale* is less virulent and offers some cross-protection against *A. marginale*, although it does not fully prevent the infection. However, its efficacy is limited: vaccine-related outbreaks caused by *A. centrale* have been reported, as well as failures to protect against virulent *A. marginale* (71). In the United States, an inactivated vaccine based on the *A. marginale* St. Maries strain has been used in the field for decades. Although this killed vaccine does not prevent infection by virulent strains, it confers sufficient immunity to protect against acute clinical disease (71). No current vaccine guarantees sterilizing immunity or universal protection across the many *A. marginale* strains, antigenic diversity among geographic isolates limits cross-protection (72).

Vaccines against *A. phagocytophilum*: Currently, there is no commercial vaccine available to prevent human granulocytic anaplasmosis or anaplasmosis in domestic animals (71). Prevention relies on tick control, as *A. phagocytophilum* is an intracellular pathogen for which no licensed vaccines exist (73). However, experimental vaccine candidates have been explored in recent years. For instance, immunizations in murine models with *A. phagocytophilum* adhesion proteins have shown partial efficacy: vaccines based on adhesin peptides (Asp14, AipA) achieved a 4–5-fold reduction in bacterial load in mice after challenge (74). Similarly, a DNA prime–protein boost strategy using conserved antigens from the type IV secretion system (VirB9-1, VirB9-2, VirB10) elicited immune responses and partial protection in mice; notably, VirB10 induced CD4^+^ IFN-*γ*^+^ T cells and reduced infection following challenge (73). In sheep, trials with the surface protein MSP4 of *A. phagocytophilum* showed limited protection, leading to “vaccinomic” approaches focused on designing more effective multiepitope chimeric antigens. By mapping protective epitopes of MSP4 and other proteins, a fusion antigen was developed that was able to block *in vitro* infection of host cells, offering a superior alternative to the use of full-length individual proteins (71).

#### 4.2.2 Local and regional strategies in Brazil

In a study conducted in Brazil, low-virulence isolates of *A. marginale* (UFMG1 and UFMG3) were evaluated as potential vaccine candidates. Nested PCR targeting the msp4 gene confirmed infection by these isolates, with the animals displaying reduced clinical signs of anaplasmosis, including lower hematocrit drops and milder anemia compared to non-vaccinated animals. These findings highlight the potential of using local low-virulence strains to enhance vaccine strategies in regions with high genetic diversity of *A. marginale* (75). The study also underscores the significance of adapting vaccine protocols to the local epidemiological context. In tropical regions like the Amazon, where the biodiversity of vectors and circulating strains is high, incorporating local isolates into vaccination programs could reduce disease persistence and improve livestock productivity. These findings align with broader efforts to develop recombinant vaccines targeting highly conserved proteins like MSP1a and MSP2. Combining live attenuated vaccines with subunit vaccines may provide a synergistic approach to tackling *A. marginale* in the Amazon and beyond.

#### 4.2.3 Challenges in vaccine development

Some limitations of available vaccines include immune evasion via antigenic variation. *A. marginale* and *A. phagocytophilum* evade the immune response through sequential variation of their major surface proteins. During chronic infection, *A. marginale* expresses antigenic variants of MSP2 and MSP3, escaping recognition by IgG2 antibodies and T cells previously primed against earlier variants (76). Similarly, *A. phagocitphylum* expresses antigenic variants of MSP2/P44 to evade immune response (76). This antigenic diversity, both among strains and within the course of a single infection, makes it difficult for a vaccine based on one or a few antigens to provide broad and lasting protection (72). The lack of cross-protection against heterologous strains has been one of the main causes of failure in previous vaccine efforts.

There are also immunogenicity limitations. Many candidate antigens do not elicit sufficiently protective immune responses on their own. For example, although MSP4 and MSP5 of *A. marginale* are relatively conserved surface proteins, they have not proven to be strong immunogens: MSP4 often fails to induce high antibody levels, while MSP5 elicits abundant antibody responses that are nonetheless non-protective (72). This underscores that immunogenicity does not necessarily equate to protection. Likewise, native membrane-associated bacterial antigens can induce protective immunity in animals, but their purified recombinant counterparts may not mimic the native conformation or context, resulting in suboptimal immune responses (72). In other words, recombinant antigens may lack key conformational epitopes or cofactors necessary to trigger adequate immunity (72). Additionally, subunit vaccines tend to focus on immunodominant but variable proteins (e.g., MSP2), which the pathogen can easily alter; therefore, researchers are exploring subdominant yet conserved antigens (such as VirB9/10 from the T4SS) as potentially more stable targets (73).

Another limitation is incomplete or skewed immune response. If the vaccine fails to trigger the appropriate cellular immune response, protection is likely to be insufficient. Anaplasmosis requires primarily a Th1-type cellular immune response (CD4^+^ T cells producing IFN-*γ* and IgG2 in cattle) to control intracellular infection (72). It has been shown that vaccination with *A. marginale* antigens combined with an appropriate adjuvant (e.g., Quil-A saponin, which promotes Th1 responses) results in high titers of IgG2, CD4^+^ T cells, and IFN-γ—correlating with clinical protection (72). In contrast, animals with an IgG1-skewed response (Th2-type humoral immunity) have developed acute disease following challenge (72). This reveals that another obstacle in vaccine development is achieving the correct immune orientation through proper formulation; an inadequate formulation (e.g., weak adjuvant) may induce insufficient or incorrect immune responses, allowing infection to persist.

One of the new approaches is using improved subunit vaccines (multiepitope recombinant antigens). Instead of single antigens, current efforts aim to design vaccines using mosaics of conserved epitopes from multiple proteins. For instance, critical peptides from adhesion proteins (Asp14, AipA, OmpA) of *A. phagocytophilum* have been assembled into a single chimeric protein (referred to as a chimeritope), allowing the inclusion of multiple protective epitopes while excluding variable or immunodistracting regions (74). This multiepitope strategy could elicit broader and more effective immune responses compared to individual antigens. In fact, a similar approach led to the development of a successful canine Lyme disease vaccine through the fusion of epitopes from various Borrelia OspC variants (74).

### 4.3 Immunotherapies and novel strategies

The study done by Noh et al. (77) highlighted the use of adhesion proteins like Msp1b and OmpA, which allows the binding of *A. marginale* to livestock’s erythrocytes, as targets for new sub unitary vaccines and immune therapies. These adhesins play a vital role in the infection process by mediating the adherence of the pathogen to the host’s cells, and eliminating this interaction could significantly reduce the infection rate. The identification of these proteins could also pave the way to the development of monoclonal antibodies that can neutralize their function, offering a new strategy to prevent the infection (77).

Another promising strategy is the use of direct mutagenesis in *A. marginale* to develop attenuated strains as a basis for vaccines. In the study of Ferm et al. (78), the gene phtcp, responsible for the pathogen-s virulence, was eliminated making a mutant strain that does not produce the disease but generates a protective immune response in bovines. This attenuated live vaccine demonstrated a high effectiveness in the protection against wild strains of *A. marginale* without causing the clinical symptoms of the disease. This technology could revolutionize the way in which vaccines against anaplasmosis are developed by reducing the severe infection risks (79).

Lastly, immune stimulation through the use of Toll Like Receptor 7 (TLR7) agonists has demonstrated to be an effective tool to activate the innate immune response in bovine livestock, providing early protection against infection by *A. marginale*. In another study by Futse et al. (80), an adjuvant along with TLR7 and saponin, which was administered to calves in an endemic region in Occidental Africa. This treatment resulted in a significant decrease in mortality and morbidity rates, also reducing the need of treatment with antibiotics. This approach offers a practical solution to protect livestock in areas where regular vaccination may not be viable, and could complement the current vaccination strategies (80).

### 4.4 Chemical, biological, and physical control strategies for vector management

Effective control of tick populations is a fundamental strategy in reducing the transmission of *Anaplasma* species, particularly *A. marginale* and *A. phagocytophilum*. Since these pathogens rely heavily on tick vectors such as *Rhipicephalus microplus* and *Ixodes ricinus*, any successful approach to control anaplasmosis must incorporate measures that directly target the vectors’ ecology and population dynamics.

In addition to *R. microplus*, multiple tick species have been implicated in the transmission of *A. marginale*. These include the three-host ticks *Dermacentor andersoni* and *D.* var*iabilis* in the United States, *Rhipicephalus sanguineus* in Israel, *R. simus* in South Africa, and the one-host tick *R. annulatus* in Israel, Central and South America, and Mexico. However, vector competence may vary significantly not only between species but also among different geographic populations of the same species (81).

Beyond vector control, an integrated approach is recommended, combining chemotherapy, immunoprophylaxis, and environmental management. Although bovine anaplasmosis causes important production losses, it has not received sufficient global attention in terms of coordinated control strategies (82). Managing grazing practices (e.g., rotational grazing), controlling arthropod vectors, and reducing contact with wildlife reservoirs through fencing are essential components (83–85). Environmental measures such as cleaning stables and proper manure disposal can further reduce vector presence (86).

Acaricides remain an important tool for tick control, but their use presents some challenges. Synthetic acaricides belong to various chemical classes, such as pyrethroids, organophosphates, amidines, macrocyclic lactones, phenylpyrazoles, and growth inhibitors (87). A study by Tosato et al. (88) demonstrated that the use of acaricides and repellents can inadvertently increase the prevalence of tick-borne diseases through a phenomenon known as overcompensation. When the tick population declines due to acaricides, the juvenile population can surge, acting as a reservoir for pathogens and facilitating disease persistence. Additionally, the use of repellents can limit the number of available hosts, increasing the likelihood of transmission between ticks through simultaneous feeding on the same host (88).

Tick control is crucial in managing anaplasmosis, as ticks serve as the primary vectors for *A. marginale*. Species such as *R. microplus* are the main transmitters, and controlling them often involves the use of tick repellents. Traditionally, these products are applied via immersion baths, spraying, or dorsal pour-on methods, along with acaricides from chemical groups like organophosphates, pyrethroids, and amidines. However, prolonged use of these products has led to the development of resistance, particularly to pyrethroids and amitraz, complicating their long-term effectiveness (89).

Given the rising resistance to chemical treatments, alternatives based on natural biocides have gained interest. A study in Ecuador evaluated the use of extracts from *Ambrosia peruviana* and *Azadirachta indica* for tick control in cattle. The results showed that a 25% solution of *A. peruviana* extract achieved a tick mortality rate of 88.33%, suggesting that natural biocides can be a sustainable and effective alternative for tick control, reducing dependence on conventional chemical products (90).

The use of entomopathogenic fungi (EPF), like *Metarhizium anisopliae* and *Beauveria bassiana*, has gained attention as an alternative for the chemical acaricides. This fungi attacks ticks by infecting their cuticula, which causes their death without the risk of producing resistance unlike the conventional chemical products. In Mexico, it has been demonstrated that EPF are effective to control resistant ticks like *R. microplus*, in laboratory and in experimental fields. This strategy is not only secure for the environment but can also be integrated with other control methods in an integrated handling of plagues (91).

One effective approach for tick control is habitat modification, which can involve methods such as controlled burns of areas where ticks are commonly found. This technique can hinder tick reproduction and survival by altering their environment. Allan (92) observed that controlled burns significantly reduced the abundance of *Amblyomma americanum* in Missouri. This method can potentially be extrapolated to other tick species that are responsible for transmitting anaplasmosis, offering a broader application for tick population management.

**Figure 3:**
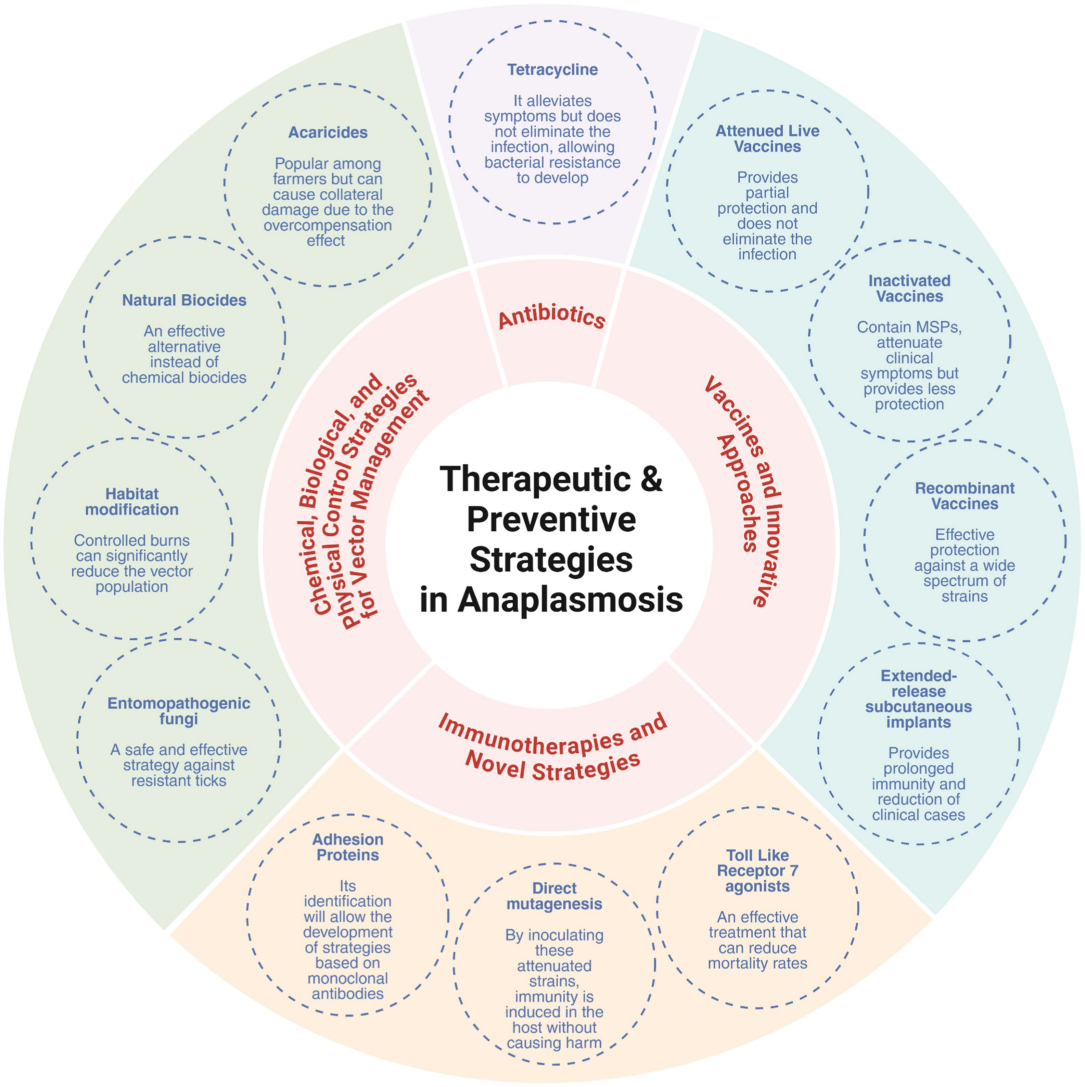
Integrated control strategies for anaplasmosis: therapeutic and preventive approaches. Created in https://BioRender.com.

Moreover, integrated vector management (IVM) strategies, combining chemical, biological, and environmental interventions, are increasingly recommended, particularly in endemic areas like the Amazon where biodiversity and wildlife reservoirs complicate tick control. These strategies not only reduce vector populations but also limit the opportunities for transmission of multiple Anaplasma species simultaneously (Figure 3).

### 4.5 Recent advances

Recent advances in molecular technologies and genetic tools have highlighted new strategies for controlling *A. marginale* infections, particularly in regions like the Amazon, where environmental factors and diverse vector-host interactions pose significant challenges.

One promising approach involves genetic therapy using CRISPR-Cas9. Although this technology has not yet been directly applied to *A. marginale*, recent studies demonstrate its potential in targeting bacterial pathogens. For instance, CRISPR-Cas phages have been used to reduce *Escherichia coli* loads in mice, showcasing the feasibility of precision bactericidal interventions (93). This strategy could potentially be adapted to specifically target *A. marginale*, offering a precise, antibiotic-free method to eliminate the pathogen and mitigate the risks associated with antimicrobial resistance.

Metataxonomic studies using 16S rRNA sequencing have also contributed to understanding the genetic diversity and dynamics of *A. marginale*. A study conducted by Makgabo et al. (94) in South Africa identified over 50 distinct genotypes of *A. marginale* across different habitats, including previously undescribed variants. These findings underscore the importance of genetic surveillance in endemic regions. Applying similar approaches in the Amazon could provide insights into the unique genetic variants circulating in the region, which may influence transmission dynamics and persistence.

Recent technological advances have opened new possibilities for the management of bovine anaplasmosis, specifically in the development of vaccine strategies and diagnosis. An example is the use of nanotechnology in vaccine design. A study in Brazil developed a nano vaccine based on Multiwalled Carbon Nanotubes (MWCNT) combined with a synthetic peptide derived from MSP1a. This platform showed promising results in murine models, inducing a balanced Th1/Th2 immune response and significantly reducing the bacteremia in immunized animals. This new approach highlights the potential of nanotechnology to overcome the limitations of traditional vaccines and offers a promising perspective for future applications in livestock, specifically in endemic regions like the Amazon (95).

Multi-omics approaches are also being explored to enhance the diagnosis of *A. marginale*. By combining genomic and transcriptomic data, researchers can identify novel biomarkers for improved diagnostic accuracy and monitor the pathogen’s genetic evolution in response to environmental and host factors. These methods hold particular promise in the Amazon, where the high biodiversity of vectors and wildlife reservoirs complicates traditional diagnostic strategies.

## 5 Future directions and challenges

### 5.1 Impact of climate change and globalization

The study by Tiffin et al. (96) highlights how climate change and globalization are affecting the distribution of ticks. Global warming has expanded the range of species like *Ixodes scapularis* in Canada, and *R. microplus* has extended its presence to new areas, making tick control more difficult. Additionally, globalization has increased the introduction of invasive species, such as *Haemaphysalis longicornis*, which poses new challenges for tick control programs. Global changes, such as climate shifts and environmental degradation, are altering ecological niches, favoring the emergence and reemergence of vector-borne diseases (97), including in forested regions like the Amazon (98).

In this environment, a vast diversity of biological species is found, many of which are arthropod vectors of pathogens or microbial species and parasites associated with diseases in humans (99). For example, rising temperatures and changing rainfall patterns may create new habitats for ticks, increasing their population and the risk of transmitting diseases such as anaplasmosis. These environmental changes not only facilitate the spread of existing diseases but may also contribute to the emergence of new pathogens. International collaboration is crucial in addressing these challenges. This underscores the need for a One Health approach, which integrates human, animal, and environmental health to effectively manage these complex and evolving threats.

### 5.2 Challenges in surveillance and control

Surveillance and control of *R. microplus* remain critical to managing anaplasmosis in the Amazon. A study by Mader et al. (100) demonstrated that underfunded and fragmented tick control programs reduce the effectiveness of disease management. Similarly, in Latin America, tropical climates support rapid tick reproduction, increasing the incidence of anaplasmosis. Also, less than 13% of sampled areas directly finance the control programs, which demonstrates the need for more coordinated and sustainable strategies (100).

In Latin America, tick control and surveillance are challenging and the main factor is the tropical weather condition inherent to this area. This condition enhances the expansion of the tick population, thus increasing the incidence of anaplasmosis. Although the use of acaricides and antibiotics help reduce anaplasmosis, tick-resistance to these strategies complicates the efforts to control this disease. Also, the implementation of control programs is difficult and unsustainable long-term.

In Peru, the government through institutions like National Service of Agricultural Sanity (SENASA) and Ministry of Agricultural Development and Irrigation (MIDAGRI) performs tick surveillance in some cities like Huancavelica, Ancash, Tumbes, Lambayeque and Puno. While these efforts help mitigate an outbreak of anaplasmosis, they are still not enough because these campaigns are performed by the government once or twice a year (101–106).

In other countries like Mexico, the approach is similar, surveillance programs once or twice a year in some regions like Jalisco, Morelos, Nayarit and Veracruz, where anaplasmosis had been previously reported and there is a risk of an outbreak of this disease (107). Similarly, the Colombian government through the Colombian Agriculture and Livestock Institute (ICA) performs tick surveillance in regions where this disease could have an outbreak (108).

In Brazil, the primary strategy for tick control heavily relies on the use of synthetic acaricides. Currently, around 250 products are marketed for tick control, and decisions regarding cattle tick management are generally made by farmers, with occasional input from veterinarians. *Rhipicephalus* (Boophilus) *microplus*, a highly invasive tick species, is prevalent in tropical and subtropical cattle-raising regions of Asia, Africa, and the Americas. In Brazil, this tick is the main vector for *A. marginale* and represents the most significant ectoparasite affecting cattle. It caused estimated losses exceeding three billion US dollars in 2014. The country’s tropical climate further complicates efforts, as it favors the proliferation of tick populations, making the control and surveillance of ticks and tick-borne diseases such as anaplasmosis particularly challenging (109).

Despite official governmental programs in other countries like Australia, Argentina, Uruguay, Mexico, and the USA, Brazil does not have an official program specifically aimed at controlling or eradicating *R*. (B.) *microplus*. However, the Ministry of Agriculture and Livestock (MAPA) has recently published a manual on the Integrated Parasite Control System (SICOPA) for managing and controlling cattle ticks. The publication, titled Avaliação Seletiva de Bovinos para o Controle do Carrapato – *Rhipicephalus microplus* (110), provides detailed guidance on selective cattle tick control.

The use of molecular techniques have been critical to improve the surveillance and management of *Anaplasma* spp., specifically in tropical regions like the Amazon, where the genetic diversity of these pathogens represents an important challenge. A recent study in small ruminants in the northeast of Brazil highlighted the high prevalence of *A. marginale*, *A. platys* and “*Candidatus A*. *boleense*” through analysis based on genes like msp4, msp1α, y 16S rRNA. These results show the complexity of the epidemiologic systems in tropical regions and the need to use multiple molecular markers for an accurate characterization of strains (111). Also, the study highlighted that the lack of systematic investigations in non-bovine species, like small ruminants, limits the comprehension of the dynamic of inter-specific transmission in environments where multiple species coexist. In the Amazon, where the environmental conditions and biodiversity favors the coexistence of multiple hosts and vetores, this lack of knowledge could compromise the implementation of effective management strategies.

The integration of advanced molecular techniques, along with active surveillance programs in small ruminants and bovines, is critical to address these challenges. This could not only improve the early detection and surveillance of *Anaplasma* spp., but also could allow the identification of new strains with zoonotic potential and adapt the interventions to local conditions of the Amazon.

### 5.3 Other emerging technologies: TickBot, gene editing, and phage therapy

Among the emerging technologies for tick control, one of the most innovative is the use of tick-catching robots, such as TickBot. This device is designed to attract and eliminate ticks from the environment, significantly reducing tick populations without relying on chemicals, making it a more sustainable strategy in the long term (112).

Gene editing using technologies like CRISPR-Cas9 has revolutionized research on various disease vectors. However, genetic editing of ticks, such as *I. scapularis*—a vector for diseases like anaplasmosis, has historically been challenging due to the unique biology of ticks, including their long-life cycle and the technical difficulties of embryonic microinjection (113). A new protocol for embryonic microinjection in *I. scapularis* has been successfully developed, and manipulation of the genome has been achieved, though the efficiency of genome editing remains low. The REMOT Control technology, which allows the injection of Cas9 complexes directly into adult ticks, has proven more effective for mutagenesis than embryonic injection. However, this technique is currently limited to genetic knockout research and does not yet allow gene insertion (113). Additionally, complementary technologies like transcriptomics and metagenomics are essential for studying how genetic manipulation affects the tick microbiome and its ability to transmit pathogens such as *A. marginale*, potentially creating new control strategies (113).

As genetic editing technologies continue to evolve, it is expected that techniques like CRISPR and other emerging approaches will offer new alternatives for controlling disease vectors. However, challenges remain regarding accessibility and practical application in the field. The use of omics tools, along with genetic manipulation, could be crucial for developing more accurate intervention strategies, especially in monitoring resistance to pathogens within the tick microbiome (114). The viability of these technologies will depend on overcoming current technical challenges and fostering more international collaboration in research and control efforts for tick-borne diseases (114).

Phage therapy, involving the use of CRISPR-Cas9 in antibacterial treatments, has shown great potential as an alternative to conventional antibiotics, particularly for controlling resistant bacteria. One of the main challenges, however, lies in the efficiency of delivering CRISPR-Cas9 to pathogenic bacteria. Although phages have been effective as vectors for delivering genetic material, the effectiveness of CRISPR systems across different bacterial species is still limited due to the variability in bacterial susceptibility to the phages used (115). Moreover, genetic editing in bacteria may lead to unintended consequences, such as the selection of more resistant strains or alterations in microbial communities, raising concerns about its long-term use in both clinical and agricultural settings.

As research progresses, it is essential to improve delivery and specificity systems in CRISPR-based therapies to reduce the risk of bacterial resistance and enhance efficacy across different pathogenic strains. Additionally, it is crucial to assess the ecological impact of these interventions, ensuring that genetic modifications in bacteria do not disrupt the microbiome associated with livestock, which could affect both animal and human health (115). Future research should focus on optimizing delivery vectors and discovering new bacterial targets for broader therapeutic applications.

## 6 Final considerations

Molecular techniques for the surveillance, diagnosis, and treatment of anaplasmosis have proven to be indispensable tools in the Amazon region and neighboring areas. Despite significant advances achieved over the years, the control, treatment, and eradication of the disease still face major challenges. These obstacles are amplified by factors such as the high diversity of vectors present in the region, the immunological complexity of the pathogen, the difficulties in implementing control strategies in extensive production systems, and the impacts of climate change, which favor vector expansion.

Although molecular techniques have enabled faster and more accurate diagnoses, the treatment of anaplasmosis remains limited by bacterial resistance and the lack of new drugs on the market. These tools could be even more effective if integrated into prevention programs that include continuous herd monitoring and proactive vector control measures. However, the economic and social realities of the region demand more accessible and sustainable solutions.

To advance in tackling anaplasmosis, it is essential to prioritize the development of new vaccines that are effective against local pathogen variants and to expand access to molecular techniques for small and medium-scale producers. The integration of control strategies based on epidemiological data, combined with public policies that encourage the adoption of good management practices, will be fundamental in reducing the impact of the disease and promoting healthier and more resilient production systems. Finally, addressing anaplasmosis in the Amazon region is not just a technical issue but also a social and economic one, requiring joint efforts from researchers, veterinarians, producers, and governments to build innovative and inclusive solutions.
